# Circulating tumor DNA tracking through driver mutations as a liquid biopsy-based biomarker for uveal melanoma

**DOI:** 10.1186/s13046-021-01984-w

**Published:** 2021-06-16

**Authors:** Prisca Bustamante, Thupten Tsering, Jacqueline Coblentz, Christina Mastromonaco, Mohamed Abdouh, Cristina Fonseca, Rita P. Proença, Nadya Blanchard, Claude Laure Dugé, Rafaella Atherino Schmidt Andujar, Emma Youhnovska, Miguel N. Burnier, Sonia A. Callejo, Julia V. Burnier

**Affiliations:** 1 Cancer Research Program, Research Institute of the McGill University Health Centre, Montreal, QC Canada; 2 Department of Ophthalmology, Centro Hospitalar e Universitario de Coimbra, Coimbra, Portugal; 3 Department of Ophthalmology, Centro Hospitalar de Lisboa Central, Lisbon, Portugal; 4 Faculdade de Medicina de Lisboa, Lisbon, Portugal; 5 McGill Academic Eye Clinic, Montreal, QC Canada; 6 Department of Ophthalmology, Centre hospitalier de l’Université de Montréal QC, Montreal, Canada; 7 Experimental Pathology Unit, Department of Pathology, McGill University, Montreal, QC Canada; 8 Gerald Bronfman Department of Oncology, McGill University, Montreal, QC Canada

**Keywords:** Circulating tumor DNA, Liquid biopsy, Uveal melanoma, Choroidal nevi, Biomarker, Mutated driver genes, In vitro study, Animal model, Clinical specimens

## Abstract

**Background:**

Uveal melanoma (UM) is the most common intraocular tumor in adults. Despite good primary tumor control, up to 50% of patients develop metastasis, which is lethal. UM often presents asymptomatically and is usually diagnosed by clinical examination and imaging, making it one of the few cancer types diagnosed without a biopsy. Hence, alternative diagnostic tools are needed. Circulating tumor DNA (ctDNA) has shown potential as a liquid biopsy target for cancer screening and monitoring. The aim of this study was to evaluate the feasibility and clinical utility of ctDNA detection in UM using specific UM gene mutations.

**Methods:**

We used the highly sensitive digital droplet PCR (ddPCR) assay to quantify UM driver mutations (*GNAQ, GNA11, PLCβ4 and CYSTLR2*) in cell-free DNA (cfDNA). cfDNA was analyzed in six well established human UM cell lines with known mutational status. cfDNA was analyzed in the blood and aqueous humor of an UM rabbit model and in the blood of patients. Rabbits were inoculated with human UM cells into the suprachoroidal space, and mutated ctDNA was quantified from longitudinal peripheral blood and aqueous humor draws. Blood clinical specimens were obtained from primary UM patients (*n* = 14), patients presenting with choroidal nevi (*n* = 16) and healthy individuals (*n* = 15).

**Results:**

The in vitro model validated the specificity and accuracy of ddPCR to detect mutated cfDNA from UM cell supernatant. In the rabbit model, plasma and aqueous humor levels of ctDNA correlated with tumor growth. Notably, the detection of ctDNA preceded clinical detection of the intraocular tumor. In human specimens, while we did not detect any trace of ctDNA in healthy controls, we detected ctDNA in all UM patients. We observed that UM patients had significantly higher levels of ctDNA than patients with nevi, with a strong correlation between ctDNA levels and malignancy. Noteworthy, in patients with nevi, the levels of ctDNA highly correlated with the presence of clinical risk factors.

**Conclusions:**

We report, for the first time, compelling evidence from in vitro assays, and in vivo animal model and clinical specimens for the potential of mutated ctDNA as a biomarker of UM progression. These findings pave the way towards the implementation of a liquid biopsy to detect and monitor UM tumors.

**Supplementary Information:**

The online version contains supplementary material available at 10.1186/s13046-021-01984-w.

## Background

Uveal melanoma (UM) is the most common primary intraocular malignancy in adults [1–3] and the second most common form of melanoma after that of the skin [4]. While rare, with an incidence of 4.2 per million [4], it is associated with a high mortality rate [3]. Patients are most commonly treated by globe-preserving plaque radiation or by enucleation for large tumors [5]. Regardless of primary treatment and effective local tumor control, approximately 50% of patients develop metastasis, primarily to the liver via hematogenous dissemination [4]. Liver metastasis is lethal in the majority of patients, with an estimated 6–12 months survival rate [4].

Predisposing risk factors for UM development include environmental exposure and intrinsic factors such as the occurrence of gene mutations (such as *GNAQ*, *GNA11*, *BAP1, CYSLTR2, PLCB4*) [6–12]. For instance, UM is characterized by constitutive activation of G protein-coupled receptor signaling, with a hotspot mutation in either G protein subunit alpha Q (*GNAQ*) or alpha 11 (*GNA11*). These mutations represent an initiating event in up to 90% of all UM cases [10, 11]. Less commonly, mutations in cysteinyl leukotriene receptor 2 (*CYSLTR2*) or phospholipase C beta 4 (*PLCB4*) have been reported [13, 14]. Pre-existing nevi in the choroid are reported as potential precursors of UM [15]. Interestingly, *GNAQ/11* mutations have also been detected in these lesions [15], suggesting that such mutations may be essential but not sufficient for malignant transformation. Choroidal nevi are the most common pigmented intraocular lesions, with a prevalence of 4.6 to 7.9% in the USA [16]. However, because these lesions are generally asymptomatic and found on ophthalmic exams performed for other reasons, it is believed that the true incidence may be much higher. Generally a nevus remains stable over time [17]; however, a rate of malignant transformation of 2, 9, and 13% at 1, 5, and 10 years, respectively, has been reported [18]. Although choroidal nevi are not biopsied, they are clinically followed for signs of growth or malignant transformation [18]. Risk factors for malignant transformation include tumor thickness greater than 2 mm, subretinal fluid, visual symptoms, orange lipofuscin pigment, tumor margin within 3 mm of the optic disc (i.e. peripapillary), ultrasonographic hollowness, and halo absence [18].

Moreover and due to the asymptomatic nature of UM, UM this disease is often detected during a routine ophthalmology examination, and its diagnosis is based on ultrasonography [4], making UM one of the few malignancies in which a biopsy is generally not used to confirm the diagnosis [19]. Detection of the classical presentations of UM generally gives rise to an accurate diagnosis; however, clinical diagnosis of nevi that have clinical risk factors that border onto malignancy becomes a challenge [20]. In addition, overlap in the size between small UM and benign choroidal nevi adds to this challenge [21]. Therefore, having a quantitative screening method is crucial to differentiate between benign choroidal nevi and small malignant melanomas, as both often share several features such as size, color, location, drusen, orange pigment, and subretinal fluid.

While biopsies are generally not used for diagnosis, they are used in UM lesions for prognostication [19]. Important prognostic factors for metastasis have been elucidated, such as chromosomal anomalies assessed by cytogenetics, gene expression profiling, and the presence of loss of function *BAP1* mutations [22]. However, tissue biopsies, aside from being invasive, provide a static picture of the tumor, neglecting spatial and temporal heterogeneity and do not sample disseminated disease, circulating tumor cells (CTCs), or micrometastasis [23, 24]. As such, UM remains challenging as it requires accurate profiling, proper interpretation of nevi (as nevi often share several features with UM [21]) and/or right risk stratification. Given the high rate of metastasis associated with this malignancy, more objective monitoring is needed to determine the best treatment options. An alternative approach that does not rely on a biopsy and that could non-invasively sample tumor-derived material would be paramount, especially to distinguish high risk nevi from small UM as well as to detect metastasis.

Liquid biopsy is a minimally invasive approach to detect and monitor disease progression, recurrence and response to treatment by investigating/assessing tumor features using different biofluids, most commonly blood, as well as other biofluids such as urine [25], saliva [26], and pleural effusion [27]. In the eye, aqueous and vitreous humors have been proposed as sources of circulating tumor DNA (ctDNA) in retinoblastoma [28, 29]. Cell-free DNA (cfDNA) has been widely studied as a liquid biopsy analyte. ctDNA can be detected within cfDNA using mutations inherent to a tumor lesion [30]. ctDNA represents as little as 0.1% of the total cfDNA [31], making its detection a challenge. Thus, a highly sensitive and specific method is needed not only for diagnosis, but also for monitoring disease progression. Digital droplet PCR (ddPCR) detects allele frequencies as low as 0.01% [32], making it suitable for ctDNA analysis.

Using this highly sensitive assay, we undertook this study to investigate the feasibility and clinical value of tracking UM-specific mutated ctDNA.

## Material and methods

### Cell lines and culture conditions

MP41 and MP46 were purchased from American Type Culture Collection (ATCC). 92.1 cells were gifted by Dr. Martine Jager (Leiden University, Netherlands) [33]. MEL270 and OMM2.5 were gifted by Dr. Vanessa Morales (University of Tennessee). OCM1 was received from the Institute of Ophthalmology and Visual Sciences (University of Valladolid). Cells were cultured in RPMI-1640 (Corning) supplemented with 10% Fetal Bovine Serum, 10 mM HEPES, 2 mM Corning glutaGRO, 1 mM NaPyruvate, 0.1% 10 U/mL penicillin and 10 μg/mL streptomycin (all from Corning), and 10 μg/mL insulin (Roche) in a 37 °C and 5% CO_2_ atmosphere.

### Animal model

Fifteen female New Zealand albino rabbits (Charles River) were used in accordance with an animal use protocol (#2018–8028) approved by the Animal Care Committee at the Research Institute of the McGill University Health Centre (RI-MUHC). Animals were randomly divided into three groups of five rabbits each: (i) group 1 (labeled G1:R1 to G1:R5), group 2 (labeled G2:R1 to G2:R5) and group 3 (labeled G3:R1 to G3:G5) (Fig. 2A). All rabbits were immunosuppressed using daily intramuscular injections of cyclosporin A (CsA at 15 mg/Kg) (Sandimmune, Novartis), starting 3 days before cell inoculation and lasting until intraocular tumor detection by fundoscopy. Following anesthesia using intramuscular injection of Acepromazine (0.75 mg/Kg), Ketamine (35 mg/Kg) and xylazine (5 mg/Kg), 1 million live 92.1 cells (in groups 1 and 2) or MP41 cells (in group 3) were inoculated into the suprachoroidal space in the right eye [34]. All rabbits were examined weekly with fundoscopy (Keeler Vantage Plus Indirect LED Binocular Opthalmoscope, lens 20D Indirect BIO lens from Volk Optical) and ultrasound (Master-Vu, Sonomed Escalon) using drops of tropicamide and phenylephrine to dilate pupils. No anesthesia or sedation were required. At fundoscopic detection of UM lesions, CsA doses were decreased to 10 mg/Kg daily in group 1, discontinued in group 2, or reduced to 5 mg/kg daily in group 3. During the experiment, all rabbits were monitored daily for CsA secondary effects (i.e. weight loss, loss of appetite, and gastrointestinal and respiratory complications). One rabbit (G3:5) was excluded from our study at week 3 due to early death following serious CsA secondary effects. Animals were euthanatized by intraperitoneal injection of pentobarbital sodium (120 mg/Kg). After enucleation, eyes were fixed in 10% phosphate-buffered formalin (Fisher). Using an DSX100 microscope (Olympus) gross pathology images were obtained. During fundoscopy examinations, tumors were measured and categorized into four categories based on basal diameter: 0 (no tumor formation), 1 (small: < 11 mm), 2 (medium: 11 to 15 mm), and 3 (large: > 15 mm).

### Patient recruitment and categorization

Forty-five participants were enrolled for this study (14 patients diagnosed with primary UM, 16 patients with choroidal nevi and 15 healthy individuals (controls)) at the McGill Academic Eye Clinic (MAEC) in accordance to an approved ethics protocol (MAEC; IRB protocol #2018–4187) approved by the Review Ethics Board of the RI-MUHC (Table 2, 3 and 4). The enrolled healthy individuals had no symptoms or personal history of cancer at the time of consent, and no family history of cancer. Blood samples were obtained following acquisition of informed consent.

Patient medical records were retrieved and used to correlate ctDNA to disease characteristics. UM patients were classified according to the American Joint Committee on Cancer (AJCC) classification of UM (Table 3) [35]. In the UM cohort, no evidence of metastasis was seen in any patient. Most of the UM patients had underwent episcleral brachytherapy (plaque radiation) as treatment, except LB36 who had not received any treatment at the time of blood withdraw (Table 3). Patients with nevi were categorized according to risk factors for UM: thickness, presence of subretinal fluid, visual symptoms, orange pigment, peripapillary, halo, and ultrasonographic hollowness (Table 4) [18].

### Blood specimen collection and sample preparation

In the animal model, following anesthesia (as stated above), 8 mL of peripheral blood was collected via the central ear artery using EDTA tubes (Becton Dickinson) at the day of cell inoculation, every 2 weeks from week 4 to 16 after inoculation and at euthanasia. For human donors, 10 mL blood samples were collected from a peripheral vein. Blood samples from all UM patients, nine patients with nevi , and eight control were collected in PAX gene Blood ccfDNA Tubes (QIAGEN/Becton Dickinson). Blood samples from the remaining seven healthy individuals and seven patients with nevi were collected in vacutainer tubes containing clot-activation additive and a barrier gel and left 60 min at room temperature to isolate serum (Becton Dickinson). Blood samples were spun at 2000 *g* for 20 min within 1 h after collection. A second spin (2000 *g* for 20 min) was performed to ensure for the elimination of contaminating cells. Resultant plasma/serum were aliquoted and store at − 80 °C until DNA isolation.

### Aqueous humor collection from rabbits

At the time of cell inoculation, at tumor formation (weeks 5–8), and euthanasia (18–19 weeks), a paracentesis was conducted to withdraw 100–300 uL aqueous humor from the anterior chamber of rabbits from groups 1 and 2 using a BD Luer-Lok 1 mL syringe (Becton Dickinson). The procedure was conducted under general anesthesia (as stated above) and following topical application of proparacaine hydrochloride ophthalmic solution (Alcon).

### DNA isolation

Genomic DNA (gDNA) was extracted from all cell lines using the QIAamp DNA Mini Kit (QIAGEN) according to the manufacturer’s instructions. cfDNA was recovered either from: (i) 3 mL cell conditioned medium: 4 × 10^5^ cells were seeded in a T25 flask (Corning), once culture reached 80% confluency, the medium was renewed. Then, after 12 h it was collected in 15 mL tubes (Falcon) and spun at 300 *g* for 5 min to eliminate contaminating cells and cell debris. (ii) 2 mL plasma/serum samples, or (iii) 100–300 μL aqueous humor samples using the QIAamp Circulating Nucleic Acid (CNA) Kit (QIAGEN). Isolated DNA was stored in AVE buffer (RNase-free water with 0.04% sodium azide-QIAGEN) in a 25 uL final volume and quantified using Qubit 2.0 fluorometer with Qubit dsDNA HS assay reagents (Supplementary Figure 2A-E and 3A) (Thermofisher Scientific).

### Analysis of mutated DNA fragments

To evaluate technical reproducibility, ctDNA was quantified from 2 mL plasma in duplicate using ddPCR. The hotspot mutations analyzed were Q209P (c.626 A > C) and Q209L (c.626 A > T) in *GNAQ*, Q209P (c.626 A > C) and Q209L (c.626 A > T) in *GNA11*, L129Q (c.386 T > A) in *CYSLTR2* and D630Y (c.1888 G > T) in *PLCB4* (Table 1).

**Table 1:** ddPCR primers and probes information for this study

	Oligos	Annealing Temperature
GNA11-F	CTTTCAGGATGGTGGATGT	
GNA11-R	ACATGATGGATGTCACGTTCT	58 °C
GNA11_A_Allele	5HEX/AC + CGC + TGG + CC/3IABkFQ	
GNA11_C_Allele	56-FAM/AC + CGC + GGGCC/3IABkFQ	
GNA11_T_Allele	56-FAM/AC + CGC + AGG + CC/3IABkFQ	
GNAQ-F	CTTGCAGAATGGTCGATGTAG	
GNAQ-R	GCGCTACTAGAAACATGATAGAG	60 °C
GNAQ_A_Allele	5HEX/CCT + T + T + G + GCCC/3IABkFQ	
GNAQ_Q209L_T_Allele	56-FAM/CCT + T + A + G + GCCC/3IABkFQ	
GNAQ_Q209P_C_Allele	56-FAM/ACCT+T + G + GGCC/3IABkFQ	
PLCb4-F	TAACAAACGGCAAATGAGTCG	
PLCb4-R	CAGCCAGCGTTCCAGAAA	55 °C
PLCB4_D630Y_G_allele	5HEX/CGA + GT + C + G + ATT + CC/3IABkFQ	
PLCB4_D630Y_T_allele	56-FAM/CGA + GT + C + T + AT+TC + CA/3IABkFQ	
CYSLTR2-F	CCTTGTATGTCAACATGTACAGC	
CYSLTR2-R	GTGAACCATTGCCAGGAAAC	55 °C
CYSLTR2_T_allele	5HEX/TTC + C + T + GA + CCGT/3IABkFQ	
CYSLTR2_A_allele	56-FAM/TTC + C + A + GA + CCGT/3IABkFQ	

Primers, probes, and synthetic DNA (i.e. gBlock used as a positive control) were designed by Integrated DNA technologies (Table 1). Serial dilutions using gBlocks were performed to obtain the minimum detection of mutant copies (Supplementary Figure 1). A 20 μL reaction mixture was prepared using 10 μL 2x ddPCR Supermix for probes (No UTP) (Bio-Rad), 900 nM forward/reverse primers, 250 nM hydrolysis probes, 1 ng DNA sample, and RT-PCR grade water (Invitrogen). DNA samples were run in triplicates, and the no-template controls and positive controls were run in duplicates. Droplets were generated using a QX200 droplet generator (Bio-Rad), and transferred into a semi-skirted 96-well PCR plate (Bio-Rad). Using a C100 thermal cycler (Bio-Rad), PCR reactions were run as follows: 10 min at 95 °C, followed by 50 cycles of 30 s at 95 °C, 1 min at 56–60 °C (optimized for each primer set (Table 1)), and 30 s at 72 °C, and finally, 10 min at 98 °C. Using a ramp rate of 2°C/s. Processed droplets were analyzed in a QX200 Droplet reader (Bio-Rad). Samples with ≤2 mutant droplets were considered as negative for ctDNA. Copies of target and percentage fractional abundance (%FA), which refers to the portion of the mutant allele frequencies over the wild type background, were generated by Quanta Soft software v 1.7.4.

### Data analysis

Our data were analyzed using levels of ctDNA (molecules/mL) and %FA. The number of ctDNA molecules was calculated following a previous reported calculation [31, 36], where the amount of copies/μL, volume added to each reaction and amount of plasma/serum are considered, as well as assuming 3.3 pg corresponded to the weight of a human haploid.

Graphs and statistical analyses were performed using Excel or Prism GraphPad Software. Spearman correlation analyses were performed to measure the degree of association between data outcomes, and comparison among groups was done by Kruskal-Wallis test. *P* < 0.05 was considered statistically significant.

## Results

### Validation of wild type and mutant cfDNA detection in conditioned medium of human UM cell line cultures

ddPCR, coupled to the noninvasive blood-based liquid biopsy, has been proposed as a promising and accurate strategy to document rare variant mutations for the monitoring of cancer development and progression [32]. By targeting well-known driver mutations that characterize UM, we performed ddPCR analyses to determine its feasibility and value for UM patient staging. We assayed serial dilutions of gBlocks that mimic several UM somatic mutations to determine the minimal detection levels of mutated copies. We were able to detect as little as 0.16, 0.08, 0.09, 0.41, 0.57 and 0.09 [copies/μL] mutated DNA for *GNAQ c.626 A > T, GNAQ c.626 A > C*, *GNA11 c.626 A > T, *and* GNA11 c.626 A > C*, *PLCβ4 c.1888 G > T,* and *CYSLTR2 c.386 T > A* mutations*,* respectively (Supplementary Figure 1).

**Fig. 1:**
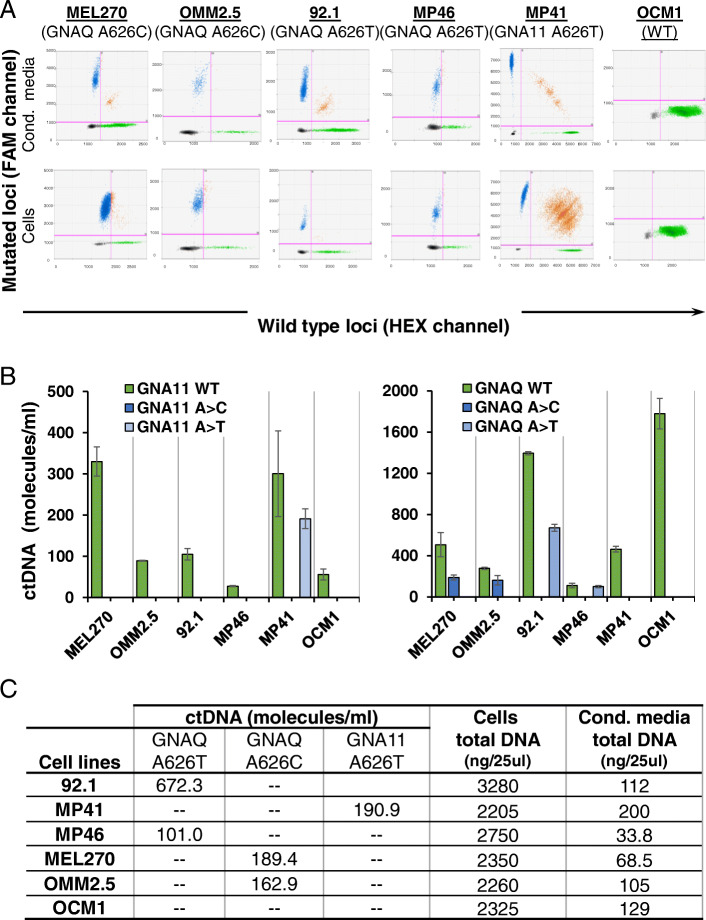
Wildtype and mutant cfDNA were accurately detected in human UM cell line conditioned medium. **A**. Representative 2D fluorescence amplitude plots of DNA extracted from conditioned medium (Cond. Media; cfDNA) and cells (genomic DNA). *GNAQ* and *GNA11* mutant DNA-positive droplets are shown in blue (FAM channel), wild type DNA-positive droplets are represented in green (HEX channel), droplets positive for both wild type and mutant targets are shown in orange, and the negative droplets are shown in black. **B**. Copy numbers of wild type and mutant cfDNA in UM cells conditioned medium. Data are shown as number of molecules per mL of medium (mean +/− SD, *n* = 3 independent experiments). **C**. Table summarizes ctDNA (molecules/mL) isolated from condicionated medium and total DNA derived from cells and conditioned medium

We first sought to determine whether we could detect cfDNA in a culture system of UM. We used six well established human UM cell lines (92.1, MP41, MP46, MEL270, OMM2.5 and OCM1) with known mutational status to set up our assay by focusing on mutations at *GNAQ* and *GNA11*. By analyzing genomic DNA (gDNA), we detected the correct mutation signature in all UM cell lines, as previously reported, which strengths the validity of our test (Fig. 1A-C, Supplementary Table 1) [37]. Notably, when we assessed DNA fragments recovered from cell-free conditioned media of these cell lines, we detected both *GNAQ/11* WT and the correct parental *GNAQ/11* mutation (c.626A > C or c.626A > T), respective to the analyzed cells, indicating the release of different amounts of fragmented DNA (i.e. cfDNA) into the culture medium (Fig. 1A-C). In addition, in conditioned media from OCM1, a known *GNAQ/11* wild type cell line, we did not detect any mutant copies of *GNAQ/11* (Fig. 1B) [38]. Together, these results indicate the feasibility of our assay to accurately and specifically detect fragmented mutant UM cfDNA targets.

### Blood mutated ctDNA levels correlated with tumor development and progression in a UM animal model

**Fig. 2:**
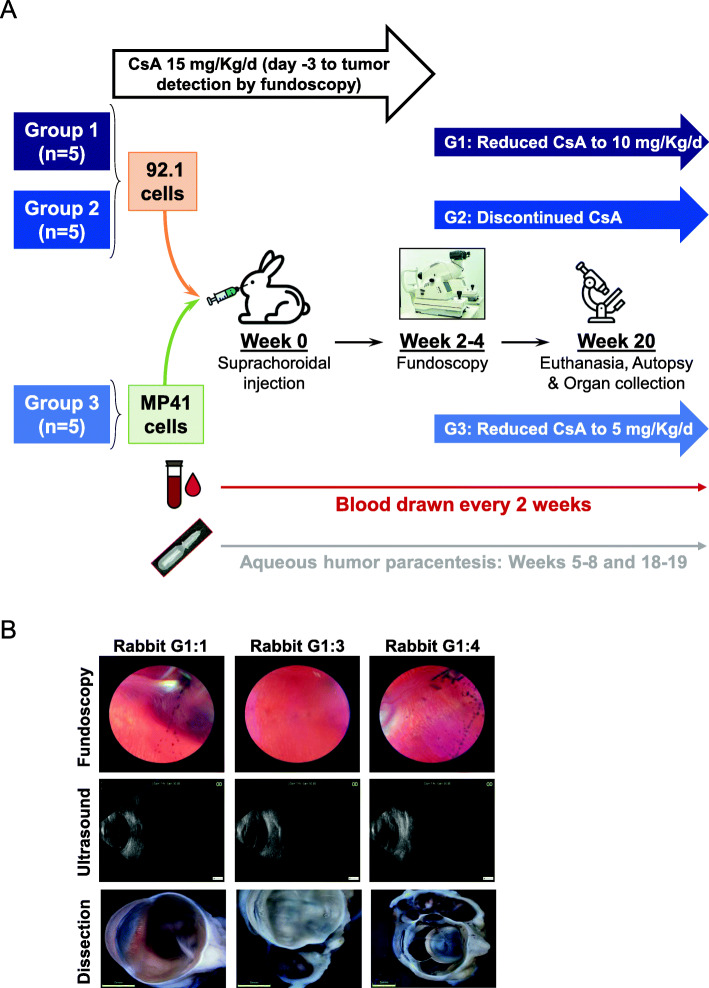
A human UM rabbit model was used to validate the detection of mutated ctDNA in liquid biopsies. **A**. Overview of the developed human UM xenograft model and animal follow-up procedures. For more details, see Material and Methods section. CsA; Cyclosporine A.** B**. Representative fundoscopy and ultrasound images taken at tumor formation, and post-mortem photographies of dissected eyes (scale bares: 5 mm)

As we had established the conditions to screen for cfDNA using the cell culture model, we applied our assay to assess the utility of *GNAQ/11* mutations in detecting ctDNA as a liquid biopsy in vivo. For this purpose, we first took advantage of a human UM rabbit model that we developed [34]. We used two cell lines with different driver mutations (i.e. 92.1 and MP41 cells harboring mutated *GNAQ* (c.626 A > T) and *GNA11* (c.626 A > T), respectively) (Fig. 2A, Supplementary Table 1). The goal is to insure the potential of screening for different UM specific mutations in vivo. During the course of the animal experiments and once ocular tumors were detected by ultrasound and fundoscopy, we adjusted the dosage of CsA to reduce the adverse effects associated with the drug (Supplementary Table 1). In addition, in the cohort of rabbits injected with 92.1 cells, CsA administration was discontinued in Group 2 to determine the potential of our assay to monitor for disease progression (i.e. tumor shrinkage) (Fig. 2A).

**Fig. 3:**
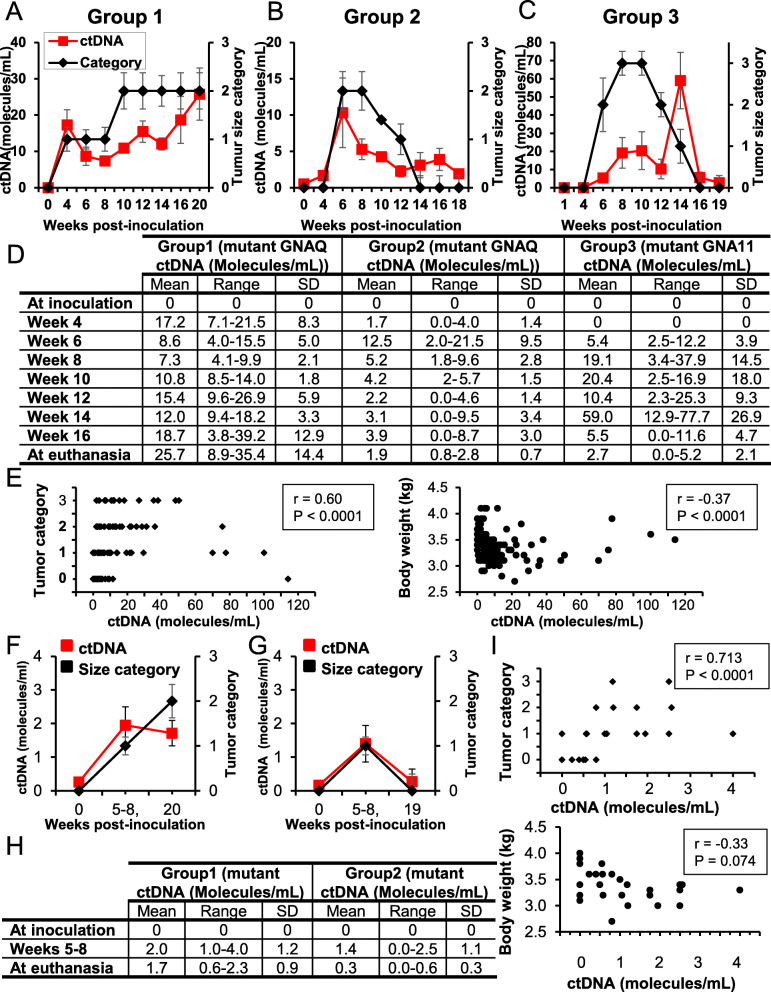
Mutated ctDNA plasma and aqueous humor levels mirrored the pattern of intraocular disease behavior in rabbits. **A-C**. Kinetics of the levels of mutated ctDNA in rabbit plasma (left Y-axis) and tumor size categories (right Y-axis) following ocular inoculation of 92.1 cells (**A** and **B; **Groups 1 and 2) or MP41 cells (**C; **Group 3). The legend for all panels is shown on the top A panel. **D.** Table shows the number of ctDNA molecules/mL in rabbit plasma at inoculation (week 0), weeks: 4, 6, 8, 10, 12, 14, 16, and at euthanasia. **E**. Mutated plasma ctDNA levels were plotted against tumor size categories (left panel) or rabbit body weight (right panel). Significant positive or negative correlations were found between ctDNA levels and size categories (r = 0.60, *P* < 0.0001) or body weight (r = − 37, P < 0.0001). **F-G**. Kinetics of the levels of mutated ctDNA in rabbit aqueous humor (left Y-axis), and tumor size categories (Cat) (right Y-axis) in animals inoculated with 92.1 cells (**F**. Group 1, **G**. Group 2). **H.** Table shows ctDNA molecules/mL from aqueous humor at inoculation, weeks 5–8, and euthanasia. **I**. Top panel: Mutated ctDNA aqueous humor levels were plotted against tumor size categories, which displayed a significant positive correlation (r = 0.713, *P* < 0.0011). Bottom panel: Mutated ctDNA levels were plotted against rabbit body weight; no correlation was found (r = − 0.33, *P* = 0.074). SD: Standard deviation

Rabbits developed detectable subretinal tumors within 4–6 weeks as judged by ultrasound and fundoscopic examinations (Fig. 2B, Supplementary Table 2). As the growth of the ocular tumors differed between rabbits, their sizes at different time points (i.e. 4, 6, 8, 10, 12, 14, and 16 weeks after cell inoculation and at euthanasia) were assessed and categorized by a clinical ophthalmologist into four categories as stated under the methods section (Fig. 3A-C). In parallel, blood samples were recovered to analyze cfDNA patterns (Fig. 2A and 3 A-D). Total cfDNA ranged from 10.1 ng to 189 ng/ 2 mL of plasma along the 20 week-study (Supplementary Figure 2A-C). In these DNA samples, and as expected, we did not detect wild type nor mutant *GNAQ/11* ctDNA of human origin at the time of inoculation (Fig. 3D**)**. In contrast, we detected mutated *GNAQ* ctDNA fragments in groups 1 and 2, and mutated *GNA11* ctDNA fragments in group 3 as early as 4 weeks and 6 weeks post-inoculation, respectively (Fig. 3 A-D), confirming the potential of our assay to screen for different UM specific mutations in vivo. Interestingly, in group 1, while ctDNA was first detected 24 days after cell inoculation, in all the animals intraocular tumors were detected clinically later, on average 31.4 days post- cell inoculation, suggesting that ctDNA was an earlier biomarker of tumor formation compared to clinical imaging. In addition, in group 2, detection of ctDNA was highest at around 6 weeks; however, levels decreased steadily and significantly afterwards until animal euthanasia (Fig. 3B and D**)**. Decreased levels of ctDNA were concomitant with the shrinkage of ocular tumors that were no longer visible (like in rabbits G2:R2 (i.e. rabbit #2 in group 2), G2:R3, and G2:R4) or very small (like in rabbits G2:R1 and G2:R5) at autopsy (Fig. 3B and D). These observations correlated with the arrest of CsA administration, suggesting the potential of our assay to monitor for disease progression**.** In group 3, where ctDNA was not detected before the 6th week, ocular tumors were detected at the 6th or 8th weeks (Fig. 3C and D), suggesting again that UM ctDNA is an earlier biomarker of tumor formation and a valuable tool to monitor UM progression. Overall, and by using Spearman correlation analyses, while no correlation was found between total DNA (not tumor specific) and ctDNA or tumor size (r = − 0.045, *P* = 0.63, or r = − 0.149, *P* = 0.39, respectively) (Supplementary Figure 2F and G), we found a significant positive correlation between ctDNA level and tumor size in all the rabbits (r = 0.60, *P* < 0.0001), and a negative correlation between ctDNA level and body weight (r = − 0.37, *P* < 0.0001) (Fig. 3E), suggesting that the correlation of blood ctDNA levels with tumor growth was specific to DNA originating from ocular UM tumor cells (i.e. ctDNA).

Taken together, these data bring strong evidence that blood biopsy screening for human mutated *GNA11/Q* ctDNA biomarkers is a valuable and feasible tool for the early diagnosis of UM and the monitoring of disease progression.

### UM-derived ctDNA detected in the aqueous humor correlated with tumor size and progression

In parallel to the analyses done on blood biopsies, we sought to determine whether UM ctDNA could be detected in the aqueous humor (AH) of grafted animals. An AH paracentesis was conducted in rabbits from groups 1 and 2, then samples were analyzed for the presence of mutated *GNAQ* ctDNA. As expected, no detectable levels of UM ctDNA were found at the time of UM cell inoculation. In contrast, we detected mutated *GNAQ* ctDNA at the second sampling (weeks 5–8, range 0–4 molecules/mL) in both groups. Interestingly, at the third sampling (weeks 18–19), we still detected mutated *GNAQ* ctDNA in the AH of rabbits from group 1 (range 1–3 molecules/mL), while we observed a sustained decrease in the AH of rabbits from group 2 with only minimal detectable traces (Fig. 3F and G). Using Spearman correlation analyses, we did not find any correlation between total DNA (not tumor specific) and ctDNA or tumor size (Supplementary Figure 2H and I). In contrast, we observed a significant and positive correlation between mutated *GNAQ* ctDNA level (tumor specific) and tumor size (r = 0.71, *P* < 0.0001) and a negative correlation between mutated *GNAQ* ctDNA level (tumor specific) and the body weight (r = − 0.33, *P* = 0.07) (Fig. 3H and I). These data suggest that the AH is another biological source of ctDNA that can be used for the management of UM patients.

### Mutant GNA11/Q ctDNA was detected in patient blood biopsy and correlated with the degree of lesion malignancy

**Fig. 4:**
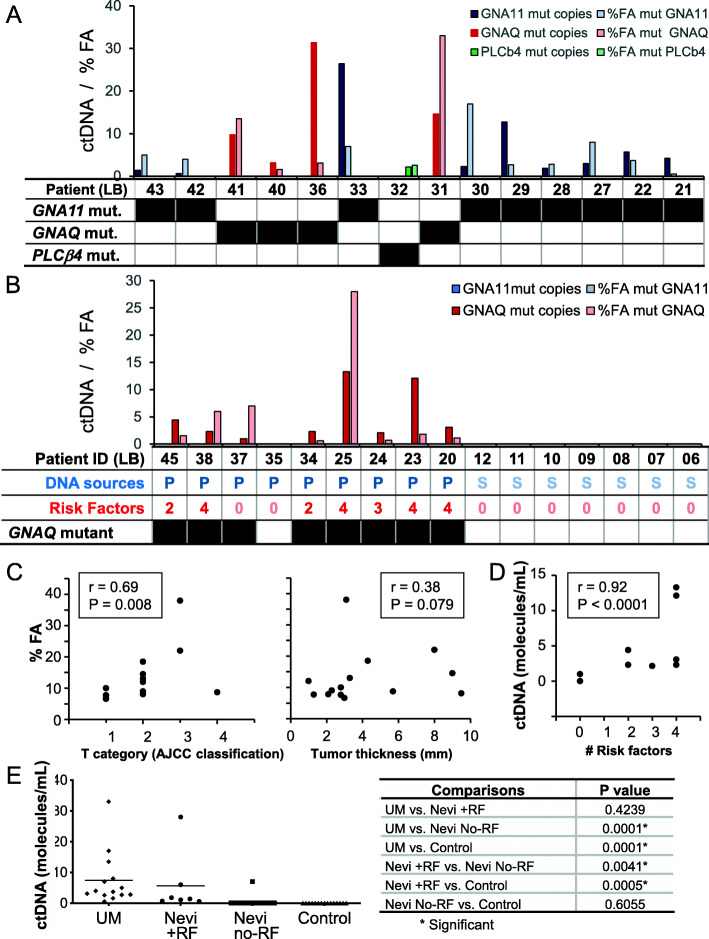
The levels of mutated ctDNA in UM patients and patients with uveal nevi correlated with the stage of disease progression and with the presence of risk factors for malignant transformation, respectively. **A**. UM patients: levels of *GNA11, GNAQ* and *PLCβ4* mutated ctDNA (molecules/mL of plasma) are shown in dark blue, red and green, respectively, and %FA of *GNA11, GNAQ* and *PLCβ4* is shown in light color (the table shows the mutation status in the analyzed loci). **B**. Nevi: levels of ctDNA molecules/mL of plasma (dark) and %FA (in light color) in patients (the table shows the mutation status in the analyzed loci). Note that in all patients, only a *GNAQ* mutation was detected. P:plasma. S:serum. **C**. Left panel: the %FA obtained with the UM samples were plotted against the T category of the AJCC staging, which displayed a significant positive correlation (r = 0.69, *P =* 0.008). Right panel: The %FA obtained with the UM samples were plotted against tumor thickness (mm); no correlation was found (r = 0.38, *P =* 0.079). **D**. The levels of mutated ctDNA in plasma samples obtained from patients with nevi were plotted against the number of risk factors in every patients, which displayed a significant positive correlation (r = 0.92, P < 0.0001). **E** Left panel: Scatter plot depicting the levels of mutated ctDNA in the plasma of UM patients and patients with nevi displaying or not risk factors for malignant transformation, and serum of healthy blood donors. Note the increased levels of ctDNA that accompany increased risk factors. The table (on the right) depicts the comparisons used and the levels of significance for differences (*P* value)

*GNAQ/11* mutations are present in more than 90% of UM cases and, although not sufficient, they are thought to be initiating events of the disease [10, 11]. As our assay was able to detect these mutations in an in vivo human UM rabbit model and correlated with disease burden, we applied this strategy to clinical samples to get more insight on its validity to screen for UM and stratify patients presenting with ocular nevi (Tables 2, 3 and 4). In addition, for cases that were negative for *GNAQ/11* mutant DNA, we verified the presence of *PLCB4* and *CYSLTR2* mutant ctDNA. As expected, all analyzed samples (from 14 primary UM patients, 16 patients with choroidal nevi and 15 disease-free healthy controls) were positive for wild type *GNAQ, GNA11, PLCβ4, CYSLTR2* ctDNA copies (Supplementary Figure 3A and Supplementary Figure 4). In addition, in contrast to patient (UM and nevi) donors, none of the 15 healthy control samples contained mutated *GNAQ/11, PLCβ4, CYSLTR2* ctDNA (Fig. 4A, B, and E, Supplementary Figure 4).

**Table 2:** Summary of patient characteristics

Total			45
UM	Number		14
	Sex	Female	7
		Male	7
	Age at blood draw (years) Mean (SD)		60.1 (15.14)
	Age at diagnosis (years) Mean (SD)		51.64 (15.35)
	Follow-up (months) Mean (SD)		103 (77.8)
	Location	Choroid	13
		Iris	1
	Tumor size (mm) Mean (SD)	Base	9.42 (3.39)
		Thickness	3.83 (2.28)
Nevus	Number		16
	Sex	Female	9
		Male	7
	Age at blood draw (years) Mean (SD)		65.6 (13.6)
	Age at diagnosis (years) Mean (SD)		62.53 (11.24)
	Follow-up (months) Mean (SD)		67.2 (61.8)
	Location	Choroid	15
		Iris	1
	Lesion size (mm) Mean (SD)	Base	3.1 (1.5)
		Thickness	1.6 (0.8)
	Risk factors		7
	No risk factors		9
Healthy individuals	Number		15
	Age at blood draw (years) Mean (SD)		30.2 (7.4)

*F/U* follow up. *SD* standard deviation

**Table 3:** UM patients characteristics

Code LB	Sex	Age (Y)	Location	ctDNA	FA	Lesion	T category, AJCC classification	FU	Rx
21	F	70	Choroid	4.3	9.5	3 × 10 × 9	2	77	P
22	F	60	Choroid	2.8	3.7	9 × 1.9 × 5.7	4	50	P
27	F	68	Choroid	3.0	8	9.7 × 9 × 3.3	2	39	P+
28	F	48	Choroid	1.9	2.8	7.5 × 5.5 × 2.1	1	260	TT
29	M	62	Iris	12.8	2.7	3.4 × 3.8 × 1.3	1	37	PR
30	M	69	Choroid	2.3	17	16 × 16 × 8	3	61	P+
31	M	85	Choroid	29.3	33	9.5 × 8.5 × 3.1	3	229	P
32	M	63	Choroid	2.1	2.6	11.5 × 12 × 2.8	1	190	P+
33	F	82	Choroid	26.4	7	2.2 × 1 × 1	2	37	P+
36	M	37	Choroid	31.6	3.1	13.3 × 10.9 × 5	2	1	P
40	F	38	Choroid	3.2	1.6	10.7 × 8.5 × 3.0	1	92	SR
41	M	71	Choroid	9.8	13.5	8.2 × 8.7 × 4.3	2	164	P
42	F	38	Choroid	0.7	4	13 × 9.5 × 2.3	2	55	P+
43	M	51	Choroid	9.0	5	8.4 × 9.1 × 2.8	1	150	P

+ = anti-VEGF treatment; *AJCC* AJCC classification; *ctDNA* (molecules/ml); *FA* percentage of fractional abundance; *FU* follow-up time (months); *Lesion* Lesion size (small diameter x large diameter x thickness) (in mm); *P* Plaque radiotherapy; *PR* Proton beam radiotherapy; *Rx* Treatment; *SR* Stereotactic radiosurgery; *T* T category; *TT* Transpupillary thermotherapy

**Table 4:** Characteristics of patients with nevi

Code LB	Sex	Age (Y)	Location	ctDNA	FA	B	TN	FU	RX	RF	Notes
06	F	73	Choroid	0	0	3	Flat	81	–	0	–
07	M	72	Choroid	0	0	NA	Flat	53	–	0	–
08	M	80	Choroid	0	0	2	Flat	141	–	0	–
09	M	79	Choroid	0	0	1.5	1.3	170	–	0	–
10	M	83	Choroid	0	0	3	Flat	182	–	0	Bilateral
11	M	64	Choroid	0	0	4	Flat	48	–	0	–
12	F	70	Iris	0	0	2.4	0.6	170	–	0	–
20	M	77	Choroid	3.1	1.1	1.8	NA	11	+	4	OP, S, F, PP
23	F	57	Choroid	12.1	1.8	NA	NA	5	+	4	T, OP, F, S
24	F	68	Choroid	4.3	0.7	NA	2.3	15	–	3	PP, F, T
25	F	35	Choroid	13.3	28	4.5	2.47	13	+	4	F, S, T, G
34	M	80	Choroid	2.3	0.6	NA	NA	9	–	2	OP, F
35	F	60	Choroid	0	0	2	Flat	12	–	0	–
37	F	47	Choroid	1	7	7	OD: Flat. OS: 1	82	–	0	Bilateral
38	F	45	Choroid	2.3	6	3.2	1.7	12	–	4	OP, F, PP, S
45	F	60	Choroid	4.4	1.8	NA	2.7	18	–	2	PP, T

+ = anti-VEGF treatment; *B* Basal diameter (mm); ctDNA (molecules/ml). *F* fluid; *FA* percentage of fractional abundance; *FU* follow-up time (months); *G* Growth; *OP* orange pigment; *PP* peripapillary; *RF* number of risk factors; *Rx* Treatment; *S* visual symptoms; *T* thickness > 2 mm; *TN* thickness (mm);

We then focused our analyses first on blood biopsies drawn from UM patients. Mutant *GNAQ, GNA11* or *PLCβ4* ctDNA copies were detected in all 14 primary UM patients. We found that these mutations were present in a mutually exclusive manner and with frequencies in the range of previously published data (i.e. 9 patients (64%) having a *GNA11* mutation (range, 0.7–26.4 molecules/mL), 4 patients (29%) having a *GNAQ* mutation (range, 3.1–31.4 molecules/mL) and 1 (7%) having a *PLCβ4* mutation (2.1 molecules/mL) (Fig. 4A, Supplementary Figure 4) [10, 11]. We did not observe any correlation between the levels of ctDNA and the age of patients or total DNA amounts (r = − 0.13, *P =* 0.48, r = 0.07, *P* = 0.34, respectively) (Supplementary Figure 3B and C).

We searched for a relationship between the levels of mutated ctDNA copies and tumor stage. We found a positive and significant correlation between the AJCC classification and %FA (r = 0.69, *P =* 0.008) (Fig. 4C (left panel) and Table 3) [39]. In addition, and although not significant, we observed a correlation between the tumor thickness and the %FA (r = 0.38, *P =* 0.079) (Fig. 4C (right panel) and Table 3). These data suggest that our assay combined to a blood biopsy is valuable to screen for patients with UM lesions and to monitor for disease aggressiveness.

We then sought to determine the pattern of mutated *GNAQ/11* ctDNA in the blood of patients presenting with premalignant choroidal nevi. In this cohort, we recruited 16 patients, and blood samples were processed to isolate either plasma (9 patients: LB20 – LB45) or serum (7 patients: LB06 – LB12) (Fig. 4B and Table 4). 

We categorized these patients according to the presence of clinical risk factors for melanoma transformation by taking advantage of a systematic classification of clinical and imaging features [40]. Of the 16 patients, 7 patients had at least 2 risk factors, and interestingly all were positive for the presence of mutated *GNAQ* ctDNA copies. Of the 9 patients with no risk factors, all, but one, were negative for ctDNA (Fig. 4B and Table 4).  The positive patient (LB37) had bilateral nevi, one of which was 1 mm thick. Notably, the levels of mutated ctDNA positively and significantly correlated with the presence of factors predisposing to nevus transformation (r = 0.92, *P* < 0.0001) (Fig. 4D).

Moreover, while the levels of mutated ctDNA in patients having nevi with risk factors did not differ compared to those in samples from the UM patient cohort, they were significantly higher when compared to levels in samples from patients having nevi with no risk factors and from healthy individuals (*P* < 0.05) (Fig. 4E). This highlights that a close follow-up of the levels of mutated ctDNA in patients with choroidal nevi may be beneficial for the early detection of potential transformation into UM lesions. Altogether, our data bring evidence that blood-based liquid biopsy screening for UM-specific mutated ctDNA may be a new tool for the early UM diagnosis, the staging of lesion malignancy and the monitoring of disease progression.

## Discussion

Although rare, UM remains the most common primary ocular cancer in adults and is associated with a high mortality rate [4]. The disease often develops asymptomatically and is diagnosed following a routine ophthalmic examination or as a result of following up patients with choroidal nevi [1–3]. This argues for the development of new quantitative tools to screen for patients at risk of developing the disease [22]. By using biological analytes, mainly blood, liquid biopsy has been investigated as a strategy to detect and monitor cancer progression, recurrence, and response to treatment [23]. Many circulating biological materials have been proposed as a readout of tumor status, of which mutated ctDNA has gained much attention [41, 42]. The presence of specific gene mutations, like in *GNAQ* and *GNA11*, were proposed as predisposing risk factors for UM development [10, 11]. As ctDNA is present at very low levels in the circulation, a highly sensitive and specific assay to detect mutated *GNAQ* and *GNA11* moieties in patient blood is needed [32]. In this study, we combined a blood-based liquid biopsy and the sensitive ddPCR assay to conduct specific UM-derived ctDNA screening [43]. We set the validity of the analysis using cfDNA from cultures of human UM cells, verified its clinical value in a human UM xenograft rabbit model, and applied it to clinical samples to correlate ctDNA levels to UM patients and patients with choroidal nevi. In summary, we bring evidence that blood biopsy permits the screening of UM-shed mutated ctDNA as a way of early diagnosis, malignancy burden staging, and disease progression monitoring.

Liquid biopsy approaches using CTCs has been proposed and applied in the context of UM, where CTCs were found in 29 out of 40 UM patients (72%) at the time of diagnosis and after treatment [44]. This is in contrast to the 100% efficiency (14 out of 14 analyzed UM patients) of UM mutant ctDNA detection we reported in our present study. In addition, ctDNA has been analyzed in UM samples using other assays (i.e. ultradeep sequencing and bidirectional-pyrophosphorolysis-activated polymerization technique), but no clinical value has been reported [45]. Our patient cohort presented a hotspot mutation in GNAQ (Q209P and Q209L), GNA11 (Q209P and Q209L), or PLCB4 *(*D630Y), which have been reported in the majority of UM cases. However, other hotspot mutations in those genes should not be discarded. Moreover, until now, the presence of ctDNA in patients with pre-malignant intraocular nevi had not been investigated. Together, this makes our study the first to investigate the presence of ctDNA using initiating UM mutations in patients with choroidal nevi and to report positive correlations between the levels of these mutated ctDNA, and both the UM staging system and the clinical risk factor classifications [18, 35, 39, 40]. Importantly, since ddPCR requires the prior knowledge of the specific point mutation [46], it is not an ideal technique in cancer types with unknown driver events or in which various point mutations can be detected. However, in UM we were able to take advantage of the high proportion of UM cases with a mutually exclusive mutation in one of the four genes assayed. ddPCR is high sensitive (i.e. 0.01% sensitivity: 1 mutant copy in 10,000 wild type gene copies) and it does not rely on sequencing efforts, making the monitoring of ctDNA in UM patients sensitive and inexpensive [31, 32, 46, 47].

In the UM rabbit model we conducted our assay anticipated in situ detection of UM lesions. Indeed, we observed an early presence of ctDNA days before standard clinical imaging techniques detected ocular tumors. This suggests that ctDNA screening in a blood biopsy may be an effective biomarker for early disease development when tumors are too small to be diagnosed by ophthalmological examination, such as fundoscopy. In addition, we observed that mutant ctDNA levels in the AH correlated with tumor burden, making it another analyte to test during patient follow-up, although accessing it is more invasive. This observation is supported by the finding that cytokine expression patterns in the AH discriminated between high- and low-risk UM patients [48]. During plaque brachytherapy implantation in UM patients, an anterior chamber paracentesis is a feasible and safe procedure that can be performed [48, 49].

In our clinical study, we were unable to match the blood biopsies to ocular tumor tissue biopsies to confirm the mutational status and validate that we screened for molecules deriving from UM tumors. This limitation was overcome by the use of the UM animal model, where the rabbits were inoculated with cells of known mutations. In these experiments, we found the corresponding parental mutations in the recovered ctDNA, respective of the cell line used (92.1 *vs.* MP41). Overall, in this animal model, our assay allowed us to monitor disease progression (Fig. 3). Notwithstanding, a well-designed study aimed to ensure matching of blood biopsy and ocular lesion genotype in clinical samples is still needed. Also, detecting ctDNA in a longitudinal study can shed light into UM dynamics and progression. A prospective study that enrolls patients from diagnosis, monitoring throughout treatment, and follow up using a single blood collection type, is needed and is currently ongoing at our center.

We found that *GNAQ* and *GNA11* mutated ctDNA is present in a mutually exclusive manner and in frequencies in the range of reported data for UM [10, 11]. Notably, while *GNA11* mutated ctDNA was more frequent in samples from UM patients, only mutated *GNAQ* ctDNA fragments were detected in samples from patients with nevi presenting with risk factors. Although further samples are still required to deepen this observation, this suggests that *GNA11* mutations are more commonly initiating events in *de novo* UM, while *GNAQ* mutations trigger malignant transformation from pre-existing nevi.

The incidence of choroidal nevi is likely underestimated, as these nevi are usually only found on ophthalmic examinations for other clinical reasons. Although nevi remain generally stable over time, malignant transformation towards melanoma increases with age and the appearance of clinical risk factors [16–18, 40, 50]. In addition, equivocal diagnosis of nevi with clinical risk factors that border onto malignancy is challenging [20]. Hence, monitoring intraocular nevi using specific ctDNA is paramount for patient follow-up. In our study, patients LB29 and LB33 were diagnosed with ocular nevi 3 and 4 years prior to the melanoma diagnosis, respectively. Furthermore, samples with undetectable levels of ctDNA may carry other hotspot mutations not included in this panel. We could not explore the correlation between ctDNA levels and thickness or base size in nevi lesions due to lack of information on medical records. However, considering clinical risk factors, our data in the nevi cohort suggest that ctDNA may indicate lesions transforming to malignant melanoma. Given the non-invasive and inexpensive nature of our testing, we propose that patients with choroidal nevus are ideal candidates to be monitored though such a liquid biopsy approach.

Preanalytical variables can influence the outcome of cfDNA [51]. Biological factors (e.g. exercise, pregnancy, inflammation, diabetes) affect the levels of cfDNA [23, 36]. Methodological variables can also impact the outcome of cfDNA [51]. For example, differences in recovery of DNA have been observed using different commercial extraction kits. In this work, the QIAamp CNA was used, which is considered as the gold standard approach [52]. In addition, higher cfDNA amounts with increased number of wild type loci copies were recovered from serum compared to plasma, likely due to the release of DNA from the lysis of white blood cells that occurs during clotting [51]. This is in line with our findings that serum was enriched in wild type *GNAQ/11* ctDNA fragments compared to plasma samples (Supplementary Figure 3G). This observation may explain the lack of detection of ctDNA in all serum samples despite having higher cfDNA levels, making the use of plasma more likely suitable for liquid biopsy-based platforms to screen for mutated ctDNA.

Half of UM patients will develop metastatic disease many years and even decades after primary ocular tumor diagnosis due to dormant micro-metastases foci [3, 4], 36 [53]. Blood-based biomarkers screening, originally designed for cutaneous melanoma, have been tested in UM patients, but have shown little promise [4, 24, 54, 55]. As metastatic patients present higher ctDNA compared to patients with primary disease, our assay may be used to screen for patients at risk of UM metastasis [56].

## Conclusion

Our study is of high importance to monitor UM patients and individuals at risk of developing the disease. Liquid biopsy is especially relevant to UM where classical tissue biopsies are generally not used for diagnosis. As a noninvasive strategy, its combination to sensitive and reliable technologies allows the monitoring of disease progression. By combining human UM cell culture and an in vivo animal model, we established the proof of principle for the validity of ddPCR to screen for specific UM mutated ctDNA in clinical UM samples. We conclude that patient plasma is an easily accessible milieu to track mutated ctDNA for the early diagnosis and staging of the patients, and the monitoring of disease progression. Further studies targeting the analysis of other UM mutations and involving a greater number of primary and metastatic UM patients, and patients with choroidal  nevi are necessary, and are currently in progress in our institution.

## Supplementary Information

**Additional file 1.**

**Additional file 2.**

**Additional file 3.**

**Additional file 4.**

**Additional file 5.**

**Additional file 6.**

## Data Availability

All data generated or analyzed during this study are included in this published article.

## Abbreviations

AJCC: American Joint Committee on Cancer
AH: aqueous humor
cfDNA: cell-free DNA
ctDN: circulating tumor DNA
*CYSLTR2*: cysteinyl leukotriene receptor 2
CI: Confidence interval
ddPCR: digital droplet Polymerase Chain Reaction
FA: Fractional abundance
gDNA: genomic DNA
*GNAQ*: Guanine nucleotide-binding protein G subunit alpha
*GNA11*: Guanine nucleotide-binding protein G subunit alpha 11
*PLCb4*: Phospholipase C beta 4
RF: Risk factors
UM: Uveal melanoma
WT: Wild type
