# Supplementary figures and images for: Circulating tumor DNA tracking through driver mutations as a liquid biopsy-based biomarker for uveal melanoma
Source: J Exp Clin Cancer Res. 2021 Jun 16;40:196. doi: 10.1186/s13046-021-01984-w (PMC8207750; doi:10.1186/s13046-021-01984-w)

## Slide 1
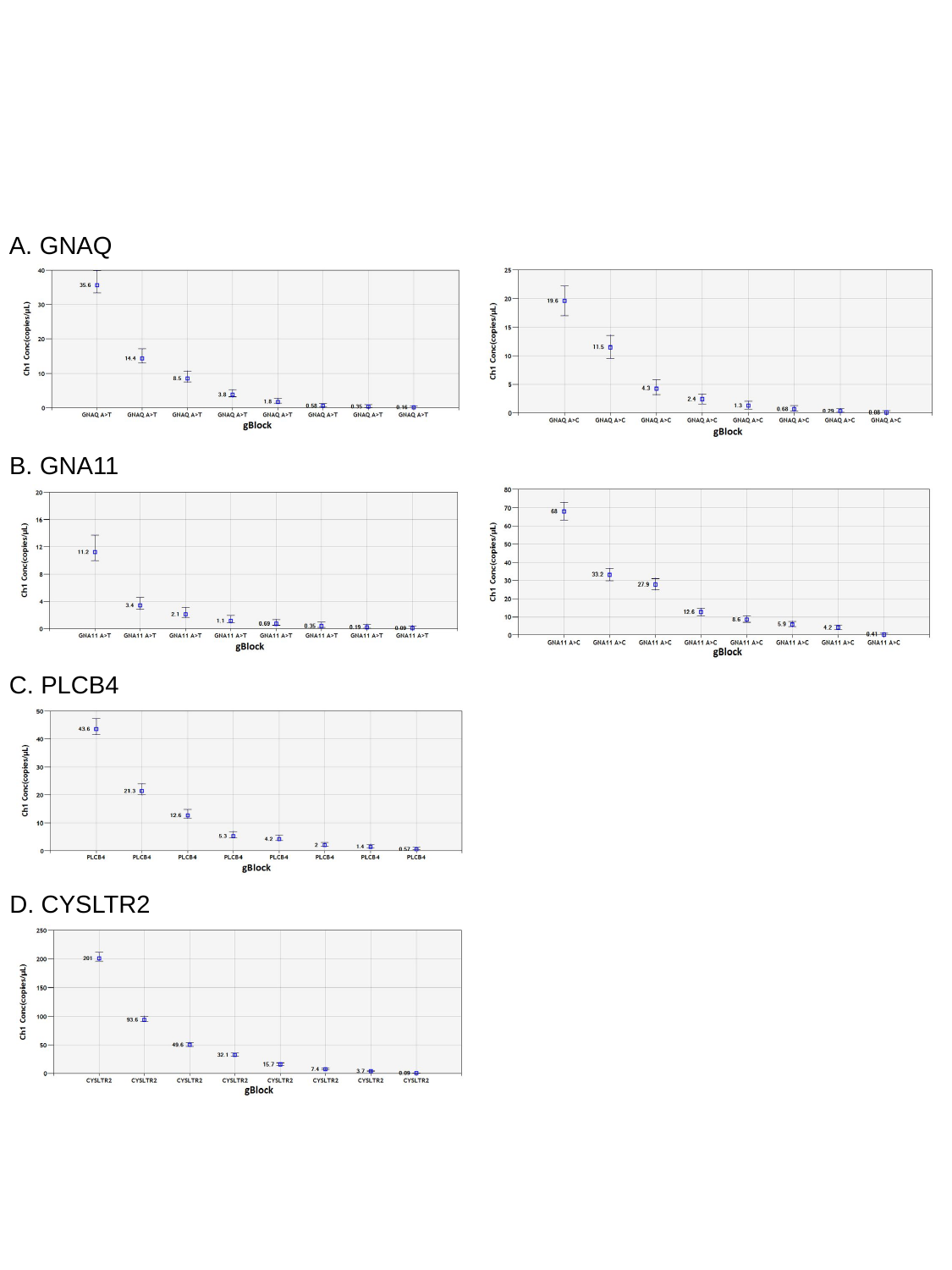

A. GNAQ
B. GNA11
C. PLCB4
D. CYSLTR2

Supplement: Supplementary file 1 — Additional file 1. [file 13046_2021_1984_MOESM1_ESM.pptx]
