# Supplementary material for: Circulating tumor DNA tracking through driver mutations as a liquid biopsy-based biomarker for uveal melanoma
Source: J Exp Clin Cancer Res. 2021 Jun 16;40:196. doi: 10.1186/s13046-021-01984-w (PMC8207750; doi:10.1186/s13046-021-01984-w)
Supplement: Supplementary file 2 — Additional file 2. [file 13046_2021_1984_MOESM2_ESM.pptx]

## Slide 1
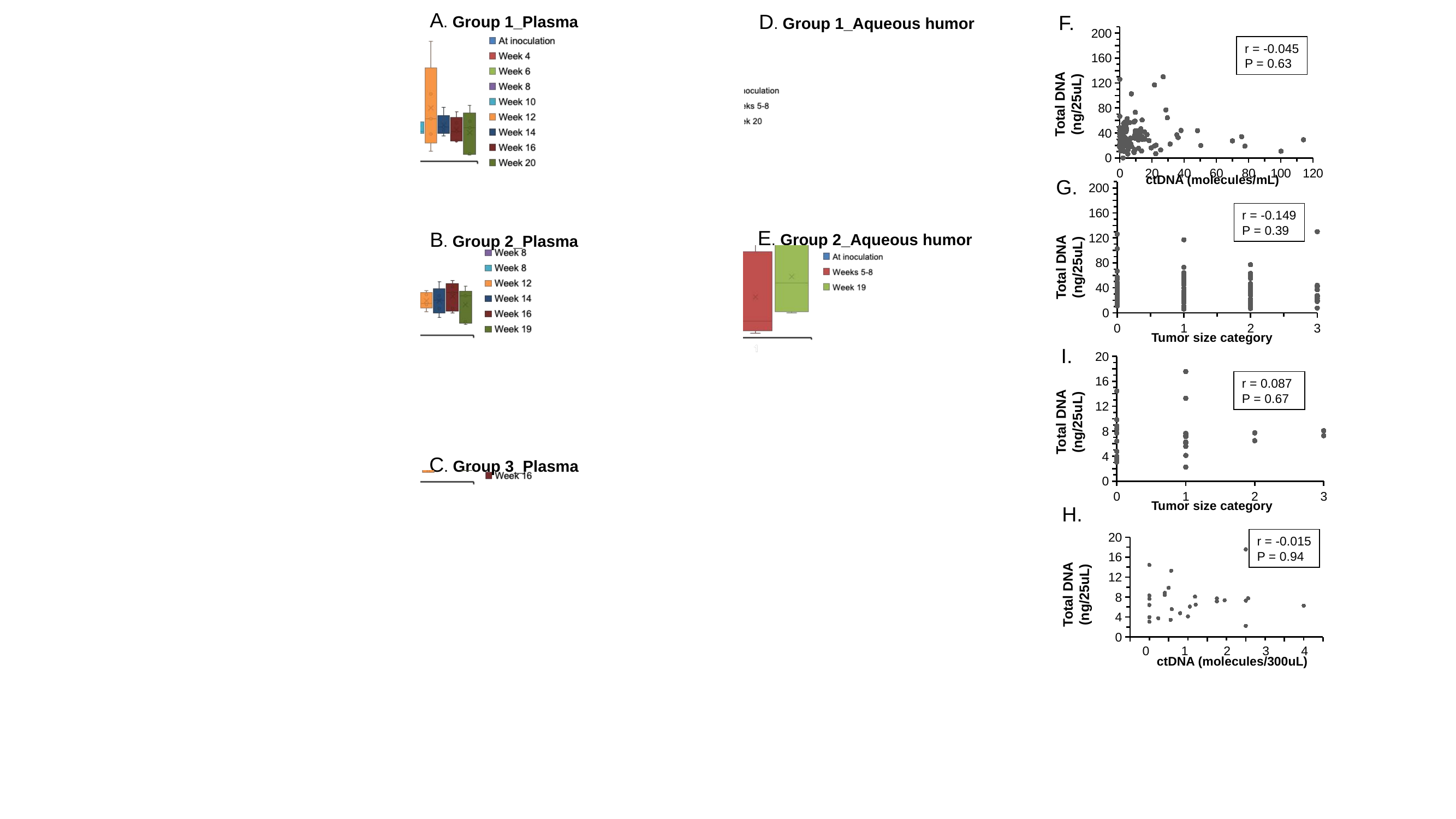

A. Group 1_Plasma
D. Group 1_Aqueous humor
F.
### Chart
| Category | |
|---|---|r = -0.045
P = 0.63
Total DNA (ng/25uL)
ctDNA (molecules/mL)
G.
### Chart
| Category | |
|---|---|r = -0.149
P = 0.39
E. Group 2_Aqueous humor
B. Group 2_Plasma
Total DNA (ng/25uL)
Tumor size category
I.
### Chart
| Category | |
|---|---|r = 0.087
P = 0.67
Total DNA (ng/25uL)
C. Group 3_Plasma
Tumor size category
H.
### Chart
| Category | |
|---|---|r = -0.015
P = 0.94
Total DNA (ng/25uL)
 0 1 2 3 4
ctDNA (molecules/300uL)
