# Supplementary material for: Circulating tumor DNA tracking through driver mutations as a liquid biopsy-based biomarker for uveal melanoma
Source: J Exp Clin Cancer Res. 2021 Jun 16;40:196. doi: 10.1186/s13046-021-01984-w (PMC8207750; doi:10.1186/s13046-021-01984-w)
Supplement: Supplementary file 3 — Additional file 3. [file 13046_2021_1984_MOESM3_ESM.pptx]

## Slide 1
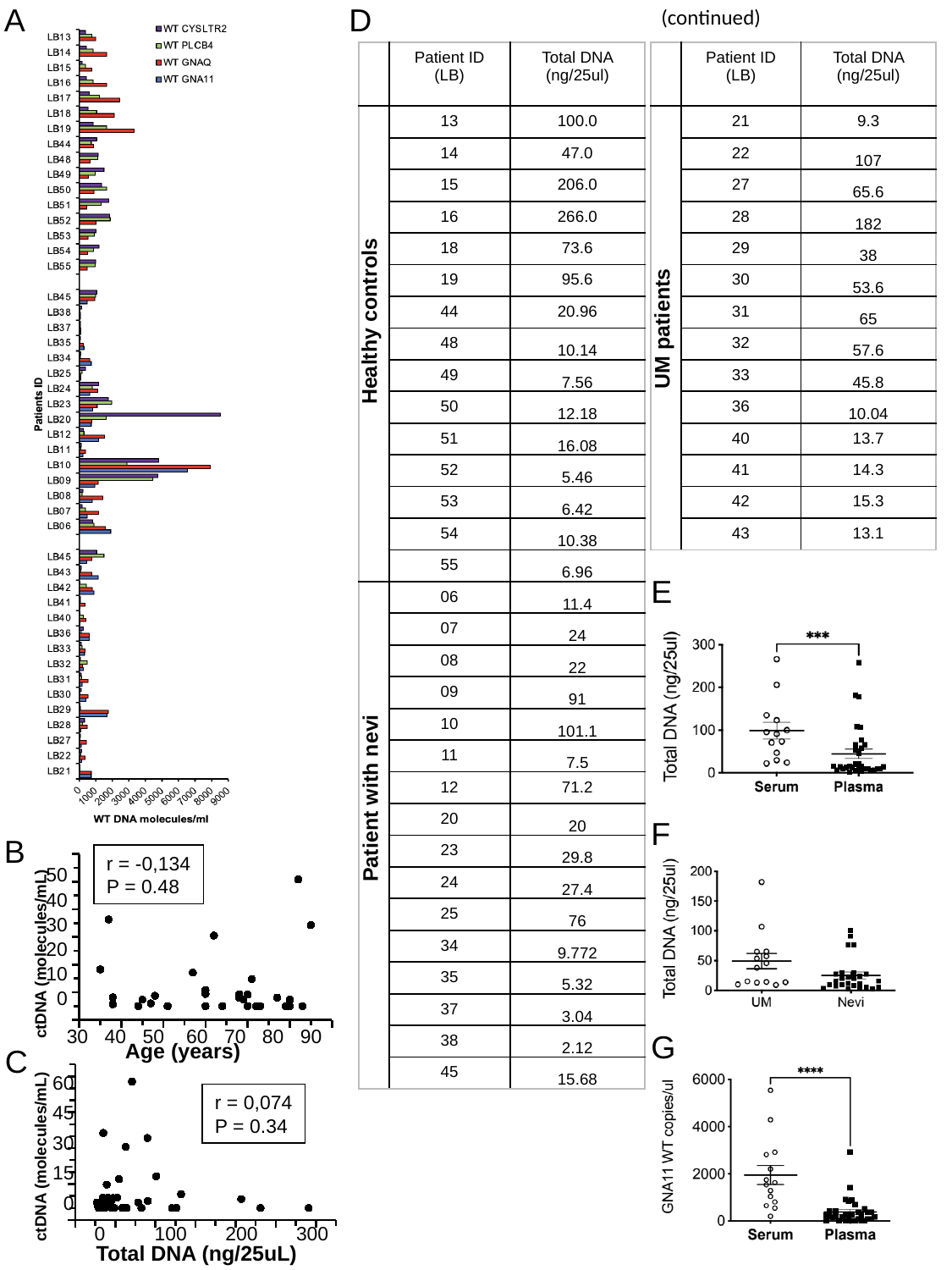

A
D
(continued)
| | Patient ID (LB) | Total DNA (ng/25ul) |
| --- | --- | --- |
| | 13 | 100.0 |
| | 14 | 47.0 |
| | 15 | 206.0 |
| | 16 | 266.0 |
| | 18 | 73.6 |
| | 19 | 95.6 |
| | 44 | 20.96 |
| | 48 | 10.14 |
| | 49 | 7.56 |
| | 50 | 12.18 |
| | 51 | 16.08 |
| | 52 | 5.46 |
| | 53 | 6.42 |
| | 54 | 10.38 |
| | 55 | 6.96 |
| | 06 | 11.4 |
| | 07 | 24 |
| | 08 | 22 |
| | 09 | 91 |
| | 10 | 101.1 |
| | 11 | 7.5 |
| | 12 | 71.2 |
| | 20 | 20 |
| | 23 | 29.8 |
| | 24 | 27.4 |
| | 25 | 76 |
| | 34 | 9.772 |
| | 35 | 5.32 |
| | 37 | 3.04 |
| | 38 | 2.12 |
| | 45 | 15.68 |
| | Patient ID (LB) | Total DNA (ng/25ul) |
| --- | --- | --- |
| | 21 | 9.3 |
| | 22 | 107 |
| | 27 | 65.6 |
| | 28 | 182 |
| | 29 | 38 |
| | 30 | 53.6 |
| | 31 | 65 |
| | 32 | 57.6 |
| | 33 | 45.8 |
| | 36 | 10.04 |
| | 40 | 13.7 |
| | 41 | 14.3 |
| | 42 | 15.3 |
| | 43 | 13.1 |
Healthy controls
UM patients
E
Patient with nevi
F
B
### Chart
| Category | |
|---|---|r = -0,134
P = 0.48
50
40
30
20
10
0
ctDNA (molecules/mL)
 30 40 50 60 70 80 90
G
Age (years)
C
60
45
30
15
0
### Chart
| Category | |
|---|---|
r = 0,074
P = 0.34
ctDNA (molecules/mL)
 0 100 200 300
Total DNA (ng/25uL)
