# Supplementary material for: Circulating tumor DNA tracking through driver mutations as a liquid biopsy-based biomarker for uveal melanoma
Source: J Exp Clin Cancer Res. 2021 Jun 16;40:196. doi: 10.1186/s13046-021-01984-w (PMC8207750; doi:10.1186/s13046-021-01984-w)
Supplement: Supplementary file 4 — Additional file 4. [file 13046_2021_1984_MOESM4_ESM.pptx]

## Slide 1
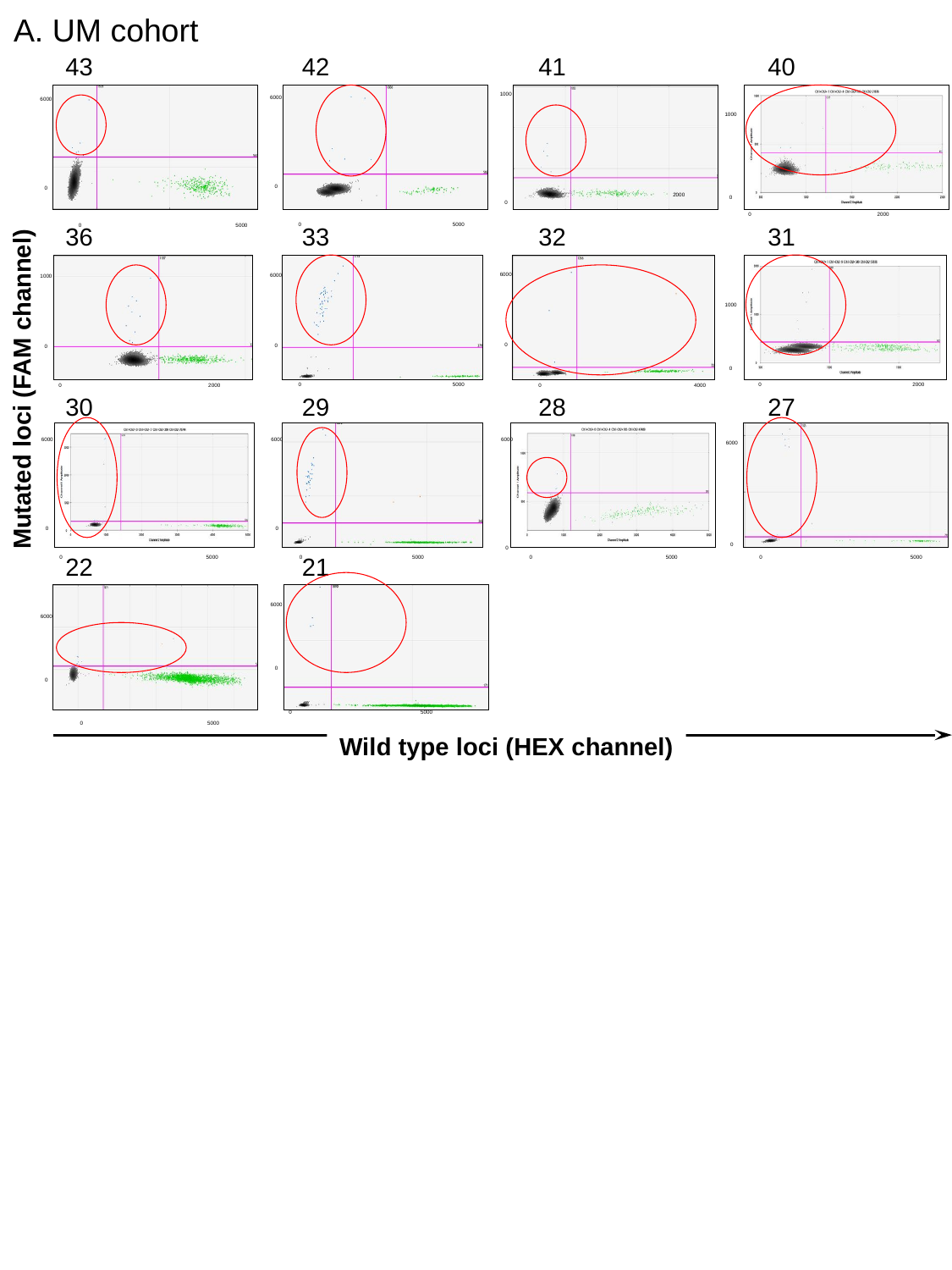

A. UM cohort
 43 42 41 40
6000
0
6000
0
1000
0
1000
0
0 2000
0 2000
 36 33 32 31
0 5000
0 5000
6000
0
1000
0
6000
0
1000
0
Mutated loci (FAM channel)
0 5000
0 2000
0 2000
0 4000
 30 29 28 27
6000
0
6000
0
6000
0
6000
0
 22 21
0 5000
0 5000
0 5000
0 5000
6000
0
6000
0
0 5000
0 5000
Wild type loci (HEX channel)

## Slide 2
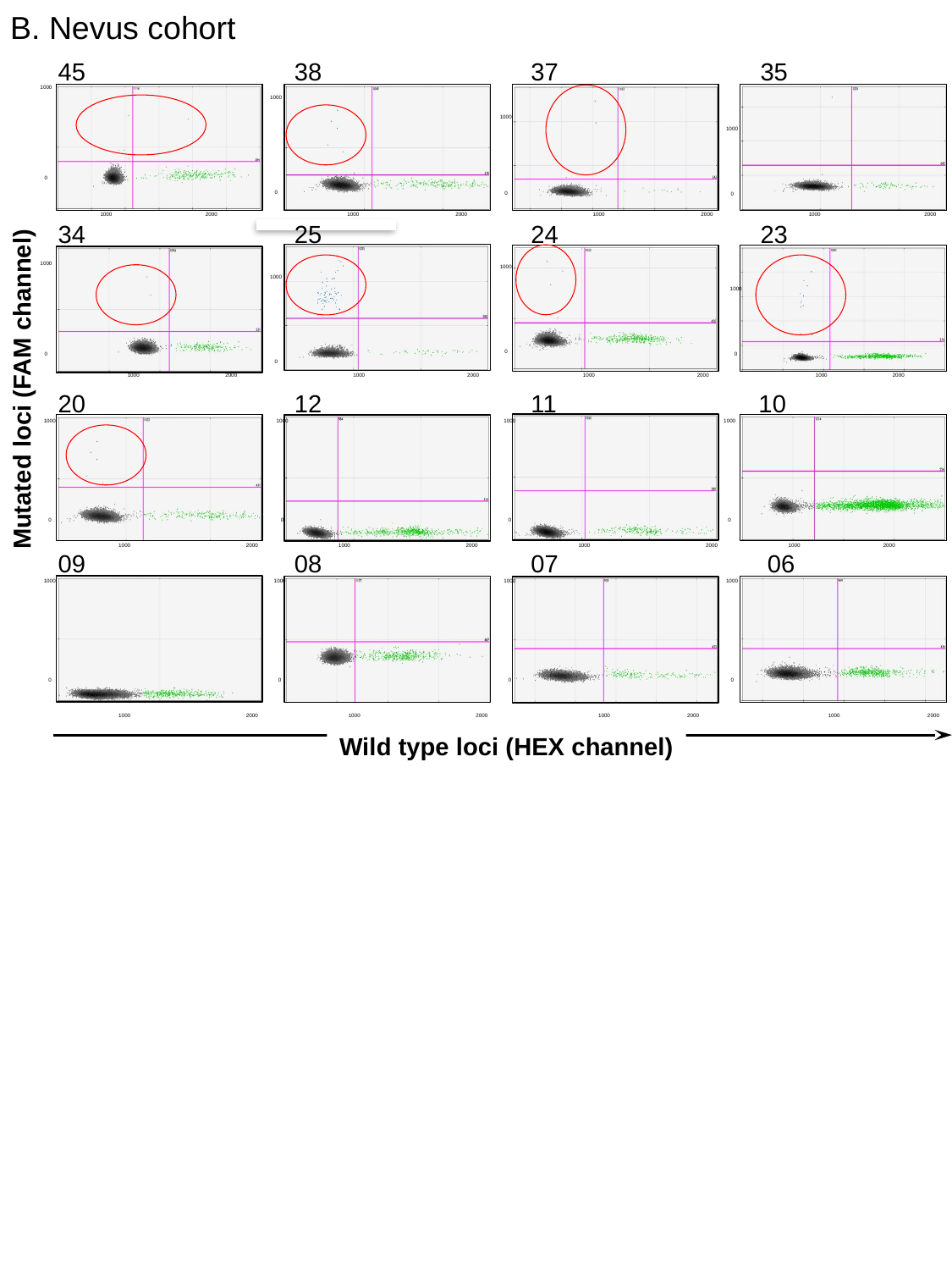

B. Nevus cohort
45 38 37 35
1000
0
1000
0
1000
0
1000
0
 1000 2000
 1000 2000
 1000 2000
1000 2000
34 25 24 23
1000
0
1000
0
1000
0
1000
0
 1000 2000
 1000 2000
 1000 2000
 1000 2000
Mutated loci (FAM channel)
20 12 11 10
1000
0
1000
0
1000
0
1000
0
1000 2000
1000 2000
1000 2000
1000 2000
09 08 07 06
1000
0
1000
0
1000
0
1000
0
1000 2000
1000 2000
1000 2000
1000 2000
Wild type loci (HEX channel)

## Slide 3
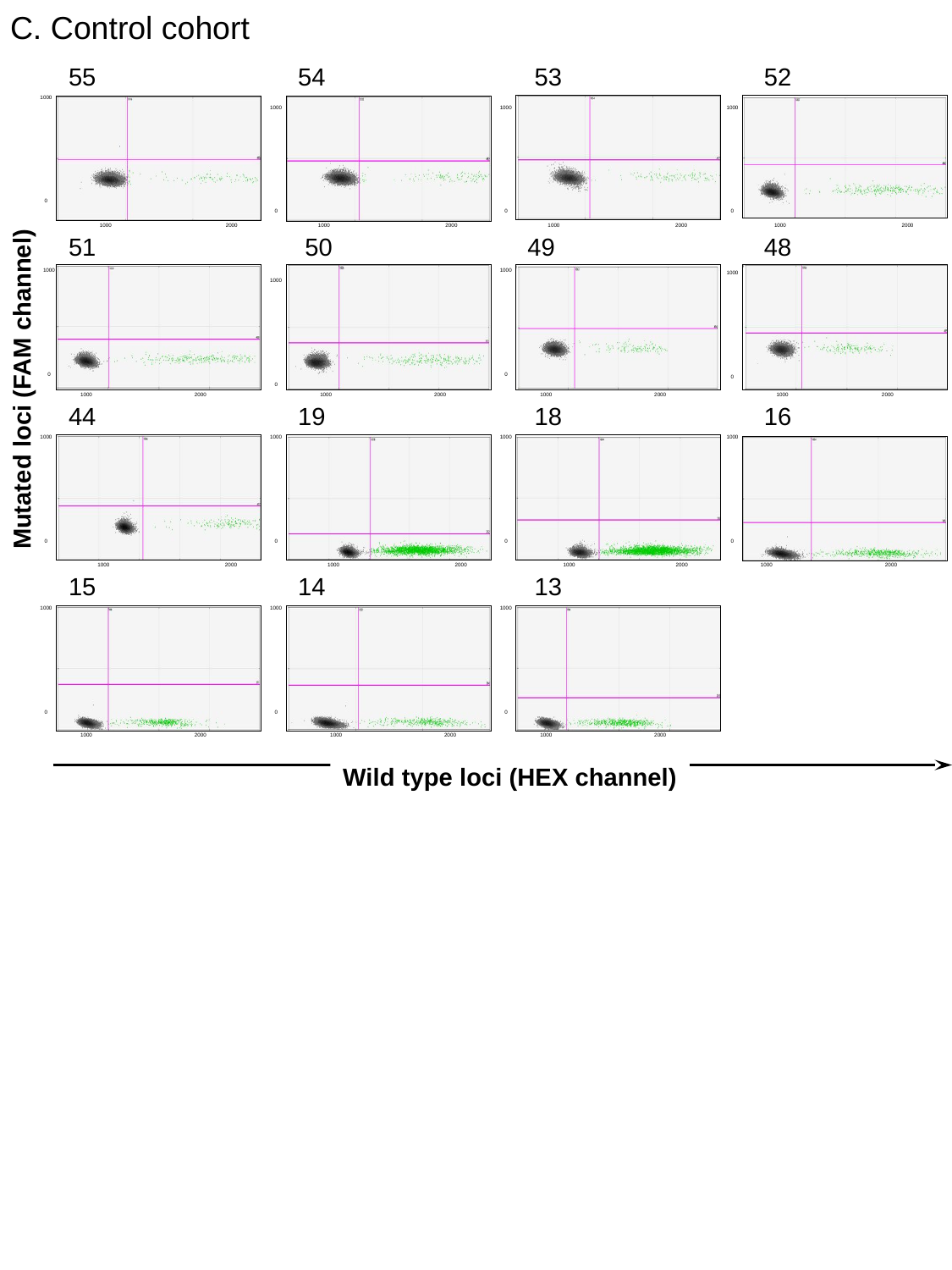

C. Control cohort
55 54 53 52
1000
0
1000
0
1000
0
1000
0
 1000 2000
 1000 2000
 1000 2000
 1000 2000
51 50 49 48
1000
0
1000
0
1000
0
1000
0
Mutated loci (FAM channel)
 1000 2000
 1000 2000
 1000 2000
 1000 2000
44 19 18 16
1000
0
1000
0
1000
0
1000
0
 1000 2000
 1000 2000
 1000 2000
 1000 2000
15 14 13
1000
0
1000
0
1000
0
 1000 2000
 1000 2000
 1000 2000
Wild type loci (HEX channel)
