# Supplementary material for: Circulating tumor DNA tracking through driver mutations as a liquid biopsy-based biomarker for uveal melanoma
Source: J Exp Clin Cancer Res. 2021 Jun 16;40:196. doi: 10.1186/s13046-021-01984-w (PMC8207750; doi:10.1186/s13046-021-01984-w)
Supplement: Supplementary file 5 — Additional file 5. [file 13046_2021_1984_MOESM5_ESM.docx]

**Supplementary materials**

**Supplementary Table 1. Description and mutational profile of used human UM cell lines.**

| **Cell line** | **Origin** | ***GNAQ/11* mutational status** |
| --- | --- | --- |
| 92.1 | PDX establish from primary tumor | *GNAQ* c.626 A>T |
| MP41 | PDX establish from primary tumor | *GNA11* c.626 A>T |
| MP46 | PDX establish from primary tumor | *GNAQ* c.626 A>T |
| MEL270 | PDX establish from primary tumor * | *GNAQ* c.626 A>C |
| OMM2.5 | PDX establish from metastatic lesion * | *GNAQ* c.626 A>C |
| OCM1 | PDX establish from primary tumor | Wild type |

* same patients
