# Supplementary material for: Circulating tumor DNA tracking through driver mutations as a liquid biopsy-based biomarker for uveal melanoma
Source: J Exp Clin Cancer Res. 2021 Jun 16;40:196. doi: 10.1186/s13046-021-01984-w (PMC8207750; doi:10.1186/s13046-021-01984-w)
Supplement: Supplementary file 6 — Additional file 6. [file 13046_2021_1984_MOESM6_ESM.docx]

**Supplementary Table 2. Summary of UM tumor formation in rabbits.**

| Mice ID | Cell line (mutation) | Time to tumor detection (weeks) | Tumor grade | Lifespan (weeks) |
| --- | --- | --- | --- | --- |
| G1:1 | 92.1 (*GNAQ*) | 5 | 3 | 20 |
| G1:2 | 92.1 (*GNAQ*) | 6 | 1 | 20 |
| G1:3 | 92.1 (*GNAQ*) | 5 | 2 | 20 |
| G1:4 | 92.1 (*GNAQ*) | 5 | 2 | 20 |
| G1:5 | 92.1 (*GNAQ*) | 5 | 3 | 20 |
| G2:1 | 92.1 (*GNAQ*) | 5 | 1 | 18 |
| G2:2 | 92.1 (*GNAQ*) | 6 | 0 | 18 |
| G2:3 | 92.1 (*GNAQ*) | 5 | 0 | 18 |
| G2:4 | 92.1 (*GNAQ*) | 4 | 0 | 18 |
| G2:5 | 92.1 (*GNAQ*) | 5 | 1 | 18 |
| G3:1 | MP41 (*GNA11*) | 5 | 0 | 19 |
| G3:2 | MP41 (*GNA11*) | 8 | 1 | 19 |
| G3:3 | MP41 (*GNA11*) | 6 | 0 | 19 |
| G3:4 | MP41 (*GNA11*) | 5 | 2 | 16 |
| G3:5* | MP41 (*GNA11*) | - | - | 3* |

* excluded from the study due to cyclosporine toxicity
